# Treatment of *Staphylococcus aureus* with environmentally relevant concentrations of triclosan activates SaeRS-dependent virulence factor expression

**DOI:** 10.1128/aac.01728-24

**Published:** 2025-06-18

**Authors:** Jeffrey M. Boyd, Erin E. Price, Franklin Roman Rodriguez, Natalie Burchat, Javiera Norambuena, Ashley L. DuMont, Victor J. Torres, Harini Sampath

**Affiliations:** 1 Department of Biochemistry and Microbiology, Rutgers, The State University of New Jersey 242612, New Brunswick, New Jersey, USA; 2 Department of Nutritional Sciences, Rutgers, The State University of New Jersey 242612, New Brunswick, New Jersey, USA; 3 Department of Microbiology, Alexandria Center for Life Science, New York University Grossman School of Medicine 171480, New York, New York, USA; 4 Department of Host-Microbe Interactions, St. Jude Children’s Research Hospital 5417, Memphis, Tennessee, USA; University of California San Francisco, San Francisco, California, USA

**Keywords:** *Staphylococcus aureus*, triclosan, virulence factor, fatty acid, two-component regulatory system, SaeRS

## Abstract

In the human pathogen *Staphylococcus aureus*, the two-component regulatory system SaeRS contributes to the expression of numerous virulence factors essential for pathogenesis. The kinase and phosphatase activities of SaeS are stimulated by several host and physiological signals, resulting in increased phosphorylation of the transcription factor SaeR and increased transcriptional activity of regulated promoters. It was recently demonstrated that the accumulation of fatty acids negatively impacts SaeS activity, decreasing titers of phosphorylated SaeP and transcriptional output. Triclosan is an effective antimicrobial that has been integrated as an ingredient in a variety of healthcare and consumer products. The chlorinated compound is recalcitrant to natural or biological transformations, resulting in environmental accumulation. At low concentrations, triclosan is a bacteriostatic inhibitor of enoyl-acetyl carrier protein reductase (FabI) of the type II fatty acid synthesis system (FASII), which is necessary for the elongation and synthesis of fatty acids. Herein, we demonstrate that the treatment of *S. aureus* with a growth-permissive concentration of triclosan alters the titers of cell-associated fatty acids and thereby functions as an activator of SaeRS. Triclosan-dependent activation of SaeRS subsequently resulted in increased transcription and expression of genes that code for virulence factors. These phenotypes are chemically reversed by the exogenous addition of oleic acid, which inactivates SaeRS, and genetically reversed by crippling the FakAB fatty acid kinase system, which generates phosphorylated fatty acids for incorporation into phospholipids. These findings present implications for the widespread use of triclosan as an antimicrobial agent in household products and its role as a persistent environmental pollutant.

## INTRODUCTION

*Staphylococcus aureus* is a human commensal bacterium and a leading cause of infectious disease-related illness and death worldwide (1). *S. aureus* infections range from relatively mild soft-tissue infections to aggressive, life-threatening infections such as sepsis, endocarditis, and pneumonia (2, 3). Treatment of these infections has become more challenging due to the increasing prevalence of antimicrobial resistance. Infections caused by antibiotic-resistant *S. aureus* result in higher mortality rates, extended hospital stays, and an increased financial strain on the healthcare system (4, 5). *S. aureus* strains have recently been isolated that are resistant to nearly all known antibiotics, including linezolid, vancomycin, daptomycin, and other “last line” drugs (6, 7). Due to the large burden that *S. aureus* continues to place on the healthcare system, an emphasis on understanding what makes *S. aureus* a successful pathogen and the design of novel, targeted therapeutics has become a priority.

The success of *S. aureus* as a pathogen is partly due to its ability to adjust and acclimate to diverse environments. To coordinate the regulation of its virulence arsenal, *S. aureus* must adapt to individual niches on and within the vertebrate host. Understanding how and when *S. aureus* alters the transcription of genes encoding virulence factors is necessary for understanding the pathogenesis. Environmental cues directing this process include changes in osmolarity, antibiotic stress, the titers of metabolites, and a range of host-associated factors. Some of these signals are sensed by *S. aureus* using two-component regulatory systems, which allow the bacterium to respond to stimuli by adjusting target gene expression accordingly (8).

*S. aureus* possesses 16 two-component systems that respond to varying stimuli to modulate gene expression (9). One of these two-component systems, the *Staphylococcus aureus* exoprotein regulator (Sae), is a master regulator of virulence factor production (10, 11). Sae is comprised of a membrane-spanning histidine kinase (SaeS), which modulates the phosphorylation status of its cognate DNA-binding response regulator (SaeR) (12). SaeS interacts with two auxiliary regulator proteins: the lipoprotein SaeP and the transmembrane protein SaeQ (13, 14). The phosphorylation status of SaeR governs its affinity for promoter DNA sequences and ultimately regulates the transcription of downstream target genes. Phosphorylated SaeR predominantly functions as a transcriptional activator of genes encoding various secreted virulence factors, including exotoxins, immune evasion factors, superantigens, adhesins, nucleases, lipases, and proteases (14, 15). It has also been shown that phosphorylated SaeR can selectively function as a transcriptional repressor of capsule polysaccharide production and certain proteases (16, 17).

Despite a detailed understanding of the Sae regulon, the precise molecular signal(s) responsible for modulating Sae output have remained unclear. SaeRS activation is modulated by several environmental stimuli, such as the neutrophil-derived signals human antimicrobial peptide (HNP-1) and calprotectin, divalent metal ions, salt concentration, acidic pH, peroxide, and beta-lactam antibiotics (15, 18–21). It has also been shown that Sae responds to self-derived signals, including the lack of cellular respiration (22). Recently, studies have demonstrated that free fatty acids modulate virulence factor production via SaeRS. The expression of SaeR targets was altered by the growth of *S. aureus* in the presence of sapienic acid from human sebum in transcriptomic and proteomic studies (23). It has also been proposed that the accumulation of intracellular fatty acids leads to altered SaeRS output in *S. aureus* strains lacking NADH hydrogenases (24). Fatty acids were implicated in decreasing SaeRS activity in studies characterizing the exogenous fatty acid kinase FakA, in which deletion of *fakA* affected the production of multiple Sae-controlled virulence factors (25–27). More recent work has demonstrated that unsaturated fatty acids and fatty acids not commonly found in Staphylococcal membranes can alter the activity of SaeRS (28). This response was dependent on the transmembrane domains of the SaeS sensor kinase protein. An increase in branched-chain fatty acids in membranes also correlated with increased titers of phosphorylated SaeR, suggesting increased SaeS kinase activity (29).

Triclosan (2,4,4′-trichloro-2′-hydroxydiphenyl ether) is a synthetic antimicrobial agent that is active against a wide range of Gram-negative and Gram-positive bacteria, including *S. aureus*. Historically, triclosan has been regarded as a biocide rather than an antibiotic and, as such, has been thought to have numerous intracellular target sites. This view has been reexamined with evidence that triclosan inhibits a specific target, the enoyl-acetyl carrier protein reductase (FabI), which is involved in the synthesis of fatty acids (30, 31). Although FabI is likely the primary cellular target, triclosan has also been shown to affect other cellular processes. For example, at high concentrations, triclosan has general membrane-disrupting activity (32–38). Because of its potent anti-bacterial properties and its ability to be readily absorbed by the gastrointestinal tract and oral mucosa, it has been included in many personal hygiene products, including soap, toothpaste, and mouthwash (typically 0.1%–0.3% triclosan by weight) (39, 40). It has also been incorporated into various household materials, including toys, fabrics, kitchen utensils, and many medical devices, such as sutures and catheters.

The findings that triclosan inhibits *S. aureus* FabI of the FASII fatty acid synthesis system and that fatty acid accumulation alters the output of the *S. aureus* SaeRS regulatory system led us to examine the effects of exposing *S. aureus* to triclosan on SaeRS activity. The results presented are consistent with the hypothesis that exposure of *S. aureus* to triclosan alters fatty acid accumulation, which stimulates SaeRS to increase the expression of virulence factors.

## MATERIALS AND METHODS

### Bacterial strains and culture conditions

Tryptic soy broth (TSB) was purchased from VWR. For solid medium (TSA), TSB was supplemented with 1.5% agar (VWR). Individual strains were grown aerobically in 2 mL of TSB in 10 mL culture tubes and shaken at 200 rpm at 37°C unless otherwise noted. For transcriptional reporter-based assays, triplicate samples of overnight cultures were subcultured and grown to an optical density A_600_ (OD_600_) of 1 and treated. Treated samples were grown for an additional 2 hours after the addition of fatty acids, BSA, or triclosan at the indicated concentrations. When selecting for plasmids or chromosomal insertions, antibiotics were added to a final concentration of 30 µg mL^−1^ chloramphenicol (Cm) or 10 µg mL^−1^ erythromycin (Erm). An amount of 10 µg mL^−1^ chloramphenicol was used for plasmid maintenance. For bacterial spotting assays, individual strains were serially diluted, and 5 µL was spotted as 10-fold dilutions on TSA plates.

### Bacterial strains and plasmids

A list of plasmids and bacterial strains utilized in this study is found in Table 1. All strains were isogenic and constructed in the community-associated MRSA strains LAC that had been cured of pUSA1, which codes for erythromycin resistance (41). All transductions were conducted using bacteriophage 80α (42). All bacterial strains were PCR-verified before use. Plasmids and PCR products were sequenced at Azenta (South Plainfield, NJ). DNA primers were purchased from Integrated DNA Technologies (Coralville, IA) and are listed in Table 2.

**TABLE 1:** Bacterial strains and plasmids used in this study

Strain identifier	Genotype	Source
JMB 1100	USA300_LAC	(43)
JMB 9891	Δ*saePQRS*	(28)
JMB 9709	*geh::saeP1_lacZ* (*sarA* RBS)	(44)
JMB 9727	*geh::saeP1_lacZ saeR::Tn* (*ermB*)	(44)
JMB 9728	*geh::saeP1_lacZ saeP::Tn* (*ermB*)	(44)
JMB 10883	*geh::saeP1_lacZ saeQ::Tn* (*ermB*)	This study, (45)
JMB 10884	*geh::saeP1_lacZ saeS::Tn* (*ermB*)	This study, (45)
JMB 1422	Newman (*saeS^P18^*)	Eric Skaar, (46)
JMB 8263	Newman *saeS^P18L^*	(47)
JMB 10923	*fabI^F204Y^*	This study
NE 822	SAUSA300_0909::Tn (*ermB*)	(45)
JMB 11601	*geh::saeP1_lacZ fabI^F204Y^* SAUSA300_0909::Tn	This study
JMB 11603	*geh::saeP1_lacZ* SAUSA300_0909::Tn	This study
JMB 13928	*fakA::Tn* (*ermB*)	This study, (45)
JMB 13931	Δ*saePQRS fakA::Tn* (*ermB*)	This study, (28, 45)
JMB 13929	*fakB1::Tn* (*ermB*)	This study, (45)
JMB 13930	*fakB2::Tn* (*ermB*)	This study, (45)

Plasmid	Construct	Source
pOS_*P1_gfp*	*saeP1* transcriptional reporter	Shaun Brinsmade
pOS-1	Cloning vector	Victor Torres
pOS_*saePQRS^P18^*	Vector carrying constitutively active SaeS allele	Victor Torres

**TABLE 2:** DNA primers used in this study

Name	Sequence	Source
Sa-Ehp-For	ACGGTATCAACGTTTGCCGGTGA	(48)
Sa-Ehp-Rev	GCTCTTTGTGCTTTACGGTGTGTTGC	(48)
Sa-Efb-For	AACAGCAGATGCGAGCGAAGG	(48)
Sa-Efb-Rev	TGCATCAGTTTTCGCTGCTGGT	(48)
coa For	TCGTTCAAGGTCCCGATTTT	(22)
coa Rev	CGGTGGGTTTGTATAATTATTGCTT	(22)
RT-hla-F	TCGTTCAAGGTCCCGATTTT	This study
RT-hla-R	CGGTGGGTTTGTATAATTATTGCTT	This study
gyrAfor	GGACGTCAACGTATTGTTGTCACT	(49)
gyrARev	CGAGCTCTGCAATTTTTTCAATC	(49)

Isolation of the *S. aureus* LAC strain containing the *fabI^F204Y^* allele was done by diluting an overnight culture of WT (JMB 1100) 1:100 and spreading 100 µL of the dillution onto a TSA plate containing 200 ng mL^−1^ triclosan. The plate was incubated at 37°C for 2 days before colonies were seen. An individual colony was streaked onto TSA with triclosan, and a single colony was isolated and maintained (JMB 10923). The *fabI* locus was amplified using PCR from WT and JMB 10923, and the PCR product was sequenced.

### Quantitative β-galactosidase assays

Quantitative β-galactosidase assays were performed as previously described (44) with slight modifications. Briefly, cell cultures (1 mL) were pelleted by centrifugation, and cell pellets were resuspended in 1 mL of Z-buffer (60 mM Na_2_HPO_4_, 40 mM NaH_2_PO_4_, 10 mM KCl, 1 mM MgSO_4_, 50 mM β-mercaptoethanol, pH 7.0), in 2 mL screw cap tubes containing 0.1 mm silica glass beads (MP Biomedicals). Cells were lysed by bead beating (two cycles, 40 s each, 6.0 m/s) using a FastPrep homogenizer (MP Biomedicals). The material was centrifuged at 13,000 × *g* for 2 min to remove whole cells and insoluble debris. Supernatant lysate (25 µL) was added to 675 µL of Z-buffer, and 140 µL of ONPG substrate (4 mg mL^−1^ [wt/vol]) was added to the samples. When the samples turned light yellow, the reactions were quenched by adding 200 µL stop solution (1 M Na_2_CO_3_), and the reaction time was recorded. The visual absorbance of the samples was measured at 420 nm using a BioTek Epoch 2 microplate reader. The corresponding culture optical densities (OD_600_) were measured, and the Modified Miller Units (specific activity) were calculated using the following equation:


$$Modified\;Miller\;Units=\frac{1000\times A_{420\;nm}}{time\;(min)\times lysate\;volume\;(mL)\times OD_{600\;nm}}$$

For anaerobic β-galactosidase assays, the reporter strains were incubated statically at 37°C in a COY anaerobic chamber. The cells were pelleted inside the chamber by centrifugation, resuspended in 1 mL of Z-buffer, and transferred to bead-containing screw-cap tubes prior to removal from the anaerobic chamber. The remaining procedure after cell lysis was carried out as described above, allowing for dioxygen exposure.

### Examining the expression of GFP using reporter constructs

Strains cultured overnight in TSB-Cm were diluted to an OD_600_ of 0.1 into 2 mL of TSB with 10 μg mL^−1^ Cm in 10 mL volume culture tubes. Once the cultures reached an OD_600_ of 1, 200 ng mL^−1^ of triclosan was added, and cells were cultured for two additional hours at 37°C with shaking at 200 rpm. One milliliter of cells was pelleted by centrifugation and resuspended in 1 mL of PBS buffer, pH 7.4. Samples of 200 µL were used for quantification of the fluorescence intensity at room temperature using a black 96-well plate. Fluorescence measurements of whole-cell liquid cultures were conducted photometrically on a Varioskan Lux plate reader (Thermo Scientific). Relative fluorescence unit measurements were taken with an excitation wavelength of 488 nm, an emission wavelength of 510 nm, and a 12 nm path length. Triplicate samples from each strain were averaged and normalized against cell density (OD_600_).

### Quantitative reverse transcriptase PCR assays

Strains were cultured overnight in TSB and subsequently diluted in triplicate to an OD_600_ of 0.1 in 2.5 mL of TSB with or without 5 µM triclosan in 10 mL glass culture tubes. Cells were incubated at 37°C with agitation until they reached an OD_600_ of 1, at which time, 1 mL of cells was pelleted by centrifugation, and cell pellets were treated with RNAprotect (Qiagen). Cell pellets were washed in 0.5 mL PBS, pH 7.4, resuspended in 100 µL 50 mM Tris, pH 8, containing 6.7 µg lysostaphin, and then incubated at 37°C with agitation for 30 minutes. The cell suspension was incubated at 65°C for 5 minutes following the addition of 200 µL of 20 mM sodium acetate, 1 mM EDTA, 0.5% SDS, and 13.4 µg lysostaphin. RNA isolation was performed as previously described (50). cDNA libraries were constructed using the High-Capacity cDNA Reverse Transcription kit (Biosystems). Quantitative reverse transcriptase PCR was performed using an Applied Biosystems StepOnePlus thermal cycler. Transcript abundances were normalized to the level of *gyrA* transcripts, which we previously found to accumulate to similar levels under different growth conditions and are only encoded once on the chromosome (51). Data were analyzed using the comparative C_T_ method (49, 52).

### Lipid extraction and quantification of free fatty acids

Strains were cultured overnight in TSB and subsequently diluted in triplicate to an OD_600_ of 1 in 5 mL TSB with or without 5 µM triclosan in 30 mL glass culture tubes. After 12 hours of growth, culture density (OD_600_) was recorded, and cells were placed on ice. For both triclosan-treated and untreated cells, an equivalent of 10 OD_600_ units of cells was transferred to 15 mL conical centrifuge tubes, and the cells were then pelleted by centrifugation (i.e., if the culture density OD_600_ of 2 was reached, then 5 mL of culture was harvested). The cell pellets were resuspended in 1.5 mL of PBS, pH 7.4, and transferred to 2 mL centrifuge tubes, and cells were pelleted by centrifugation. Cell pellets were resuspended in 300 µL of a 1% (vol/vol) Triton X-100 chloroform solution in 2 mL screw-cap tubes containing 0.1 mm silica glass beads (MP Biomedicals). Cells were lysed by bead beating (two cycles, 40 s each, 6.0 m/s) using a FastPrep homogenizer (MP Biomedicals). The material was centrifuged at 13,000 × *g* for 10 min to remove whole cells and insoluble debris. Equal amounts of the organic (lower) phase of each sample were collected and transferred to a 1.5 mL centrifuge tube, and lipids were air-dried at 50°C for 1 hour in a fume hood to remove trace chloroform. Dried lipids were dissolved in 100 µL of assay buffer and vortexed extensively for 5 min. The concentration of free fatty acids from each sample was determined fluorometrically using a free fatty acid quantitation kit from Sigma-Aldrich (catalog number MAK044). Fluorescence measurements were taken at room temperature using a black 96-well plate on a Variskan Lux microplate reader (Thermo Scientific) with an excitation wavelength of 535 nm, an emission wavelength of 587 nm, and a 12 nm path length.

### Quantification of individual free fatty acids

Cells were grown and harvested as discussed for lipid extraction. Lipids were extracted by a modified Folch method and separated by thin-layer chromatography, as previously described (53, 54). Briefly, cells were lysed with glass beads in chloroform:methanol (2:1), and the extracted lipids were phase-separated by the addition of acidified saline, followed by centrifugation. The lower organic phase was dried and reconstituted in chloroform:methanol (2:1), spotted onto Silica Gel 60 chromatography plates (Sigma-Aldrich, St. Louis, MO), and developed in heptane:isopropyl ether:acetic acid (60:40:3) until the solvent front reached 1 cm from the top of the plate. TLC plates were sprayed with 0.2% (wt/vol) 2′,7′-dichorofluorescein (Sigma-Aldrich) in ethanol, and bands were visualized under UV light (321 nm). Bands corresponding to free fatty acids were identified using a known standard, scraped, and collected for further analysis. Lipids were methylated in 14% BF_3_ in MeOH (Sigma-Aldrich), heated to 100°C, and extracted in hexane. The lipid fraction was dried, reconstituted in hexane, and loaded onto the Gas Chromatography and Mass Spectrometry system (Agilent Technologies, Santa Clara, CA, United States) using an autosampler. MassHunter Data Acquisition software and MassHunter Quantitative Analysis software were utilized for peak analyses. Peak identity was confirmed by a National Institute of Standards and Technology (NIST) library search. The area under the curve values were generated by MassHunter and converted to concentration using standard curves generated for each fatty acid.

### Purification of human neutrophils

LeukoPaks containing peripheral white blood cells from anonymous donors were obtained from the New York Blood Center. LeukoPak samples were gently mixed with an equal volume of 0.9% NaCl with 3% dextran to allow for the sedimentation of the red blood cells. The top fraction of cells containing polymorphonuclear cells (PMNs) and peripheral blood mononuclear cells (PBMCs) was transferred to new 50 mL conical tubes and washed once with PBS. Cells were resuspended in Hanks’ Balanced Salt Solution (ThermoFisher, Waltham, MA), then layered on top of Ficoll (Ficoll-Paque PLUS, GE Healthcare, Chicago, IL). Cells were then centrifuged for 30 min to separate cell types by density. The supernatant was removed, and the PMN pellet was washed in PBS. The PMNs were then resuspended in Gibco ACK Lysis Buffer (ThermoFisher, Waltham, MA) to remove any remaining red blood cells. Purified PMNs were resuspended in Roswell Park Memorial Institute (RPMI) medium with 0.1% human serum albumin (HSA, SeraCare Life Technologies, Inc., Milford, MA) and 10 mM HEPES buffer.

### Cytotoxicity assay with human neutrophils

Triplicate samples of overnight *S. aureus* cultures in TSB were sub-cultured into TSB medium by diluting the cultures 1:100 into 5 mL of TSB medium in 30 mL culture tubes with shaking at 37°C. Strains were grown to an OD_600_ of 1, 200 ng mL^−1^ triclosan or 200 µM arachidic acid was added to some of the treated cultures, and the cells were grown an additional 2 hours (mid-log phase). The cells were pelleted by centrifugation, and the supernatant was filtered through a 0.45 µm syringe filter to remove any remaining cells or particulates from the conditioned media. The supernatants were stored at −80°C prior to use.

Following purification, primary human PMNs were counted and normalized to 4 × 10^6^ cells/mL in RPMI (without phenol red) with 0.1% HSA and 10 mM HEPES. The cells were plated in flat-bottomed 96-well tissue-culture plates at 200,000 cells per well (50 µL per well). PMNs were incubated with *S. aureus* culture supernatants starting at 50% culture supernatant (50 µL). The culture supernatants were titrated on the PMNs by serially diluting the supernatants twofold in TSB. PMNs were incubated with the culture supernatants for 1 hour at 37°C with 5% CO_2_. Cellular metabolism was evaluated by adding 10 µL of CellTiter (Promega), incubating the mixture at 37°C with 5% CO_2_ for 1.5 hours, and then reading the plates with a PerkinElmer EnVision 2103 Multilabel Reader at an absorbance of 492 nm. The percent of dead cells is calculated by subtracting out the background (healthy cells plus PBS) and normalizing to Triton X-treated cells, which are set at 100% dead.

### Immunoblot

*S. aureus* culture supernatants used for the neutrophil cytotoxicity assays were precipitated with 10% trichloroacetic acid (TCA) overnight at 4°C. The samples were then centrifuged at 15,000 rpm, 4°C, for 15 min, and the supernatant was aspirated. The remaining pellet was washed with ice-cold 100% ethanol for 30 min at 4°C. The samples were centrifuged at 15,000 rpm, 4°C, for 15 min. The supernatant was aspirated, and the pellets were allowed to air dry for 1–2 hours at room temperature. The pellets were then resuspended in 8 M urea, incubated at room temperature for 30 min, and then mixed with SDS sample loading buffer. The samples were run on 10% polyacrylamide gels followed by transfer to nitrocellulose membranes, which were then blocked for 1 hour at room temperature on a rocker with PBS with 0.1% Tween 20 and 5% milk. The primary antibodies (anti-LukF mouse monoclonal, clone 2-4.2.6) were diluted 1:5,000 in PBS with 0.1% Tween 20 and 5% milk and incubated with the membrane overnight at 4°C on a rocker. The blots were washed with PBS with 0.1% Tween 20, then incubated with goat anti-rabbit or goat anti-mouse Alex Fluor680 secondary antibodies diluted in PBS with 0.1% Tween 20 and 5% milk for 1 hour at room temperature on a rocker. The blots were washed with PBS with 0.1% Tween 20 and then imaged using the Odyssey CLx Imaging System. The relative fluorescence was quantified with the LI-COR Image Studio Software as done previously (55).

## RESULTS

### The transcriptional activity of the *saeP1* promoter is modulated by long-chain free fatty acid titers

The *S. aureus sae* locus is comprised of four open reading frames (*saePQRS*), which have two promoter sequences denoted as P1 and P3. The first promoter, P1 (called *saeP1*), lies upstream of *saeP* and transcribes *saePQRS*. The *saeP1* promoter is autoinduced by SaeR and is, therefore, responsive to the phosphorylation status of SaeR (14). We created an *S. aureus* USA300_LAC strain containing the *saeP1* promoter driving expression of *lacZ*, which codes for β-galactosidase. We integrated the construct within the *geh* locus to not alter SaePQRS activity (44). Ericson et al. demonstrated that transcript levels of SaeRS-regulated genes significantly decreased after adding exogenous long-chain fatty acids (27). Similarly, a recent study from DeMars et al. demonstrated that coagulase (*coa*) promoter activity, used as an indicator of SaeRS activity, was significantly decreased in the presence of long-chain free fatty acids (28).

**Fig 1:**
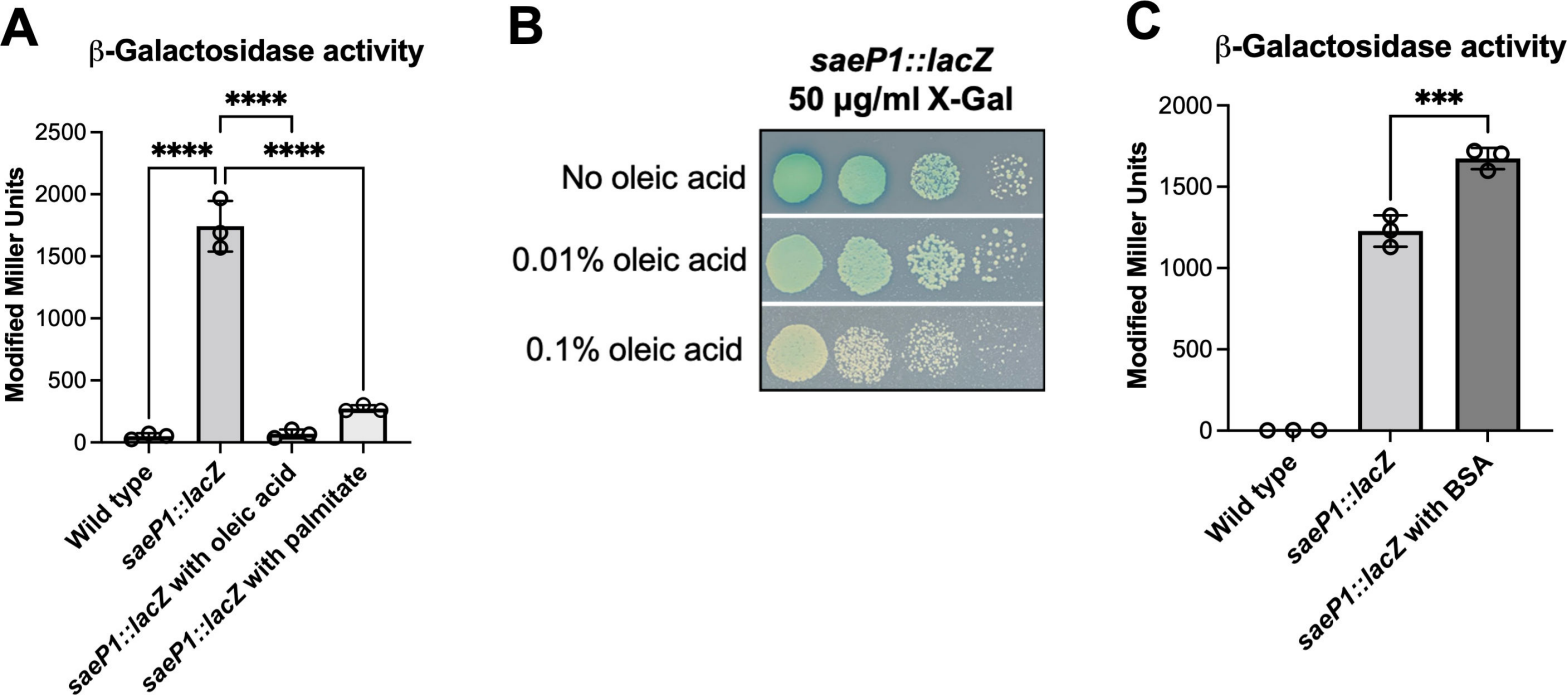
Long-chain free fatty acids influence *S. aureus saeP1* promoter activity. (**A**) Saturated and unsaturated long-chain fatty acids inhibit *saeP1* promoter activity. Beta-galactosidase activity was quantified in the wild-type (JMB 1100) and *saeP1::lacZ* (JMB 9709) strains cultured until logarithmic phase in TSB medium supplemented with and without 0.1% oleic acid or 100 µM palmitate. (**B**) Overnight cultures of the *saeP1::lacZ* strain were serially diluted (10^−2^ through 10^−5^) and spotted on TSA plates containing 50 mg mL^−1^ X-gal and the indicated concentrations of oleic acid. (**C**) Stimulatory impact of fatty acid depletion on *saeP1* promoter activity. Beta-galactosidase activity was quantified in the *saeP1::lacZ* strain cultured to logarithmic growth phase in TSB supplemented with or without 10 mg mL^−1^ fatty acid-free bovine serum albumin (BSA). The data depicted in panels A and C are the average of biological triplicates, with the standard deviations shown. Sample means were compared using one-way ANOVA, and pair-wise comparisons were conducted using *post hoc* Tukey’s multiple comparison tests. Significance levels are as follows: *** and **** represent *P*-values of ≤0.001 and ≤0.0001, respectively. Representative images are displayed in panel B.

To determine whether the presence of long-chain free fatty acids impacts the transcriptional activity of the *saeP1* promoter, the *saeP1::lacZ* strain was cultured in media supplemented with oleic acid (18:1) or palmitic acid (16:0). These treatments were chosen because they represent the highest abundance of unsaturated and saturated long-chain fatty acids found on human skin (56, 57). Exogenous treatment of *S. aureus* with either oleic acid or palmitic acid significantly decreased *saeP1* transcriptional activity compared to untreated cells (Fig. 1A). We also examined *lacZ* expression driven by the *saeP1* promoter on solid medium containing X-gal. In this case, a decrease in the blue color intensity of the *saeP1::lacZ* strain can be visualized as oleic acid was titrated into the medium at increasing concentrations (Fig. 1B). These results demonstrate that long-chain free fatty acids decrease *saeP1* promoter activity.

If supplementing the media with free fatty acids decreases *saeP1* promoter activity, reducing levels of fatty acids should conversely stimulate the *saeP1* promoter. Fatty acid-free bovine serum albumin (BSA) has a high affinity for fatty acids, and supplementing the growth medium with this BSA lowers titers of fatty acids in the medium, shifting the equilibrium and resulting in lower intracellular fatty acid concentrations (27). Indeed, we observed a significant increase in β-galactosidase activity in the *saeP1::lacZ* strain when the media were supplemented with fatty acid-free BSA (Fig. 1C). These results suggest that *saeP1* transcriptional activity is stimulated upon fatty acid depletion.

### Triclosan treatment increases SaeRS regulatory output

**Fig 2:**
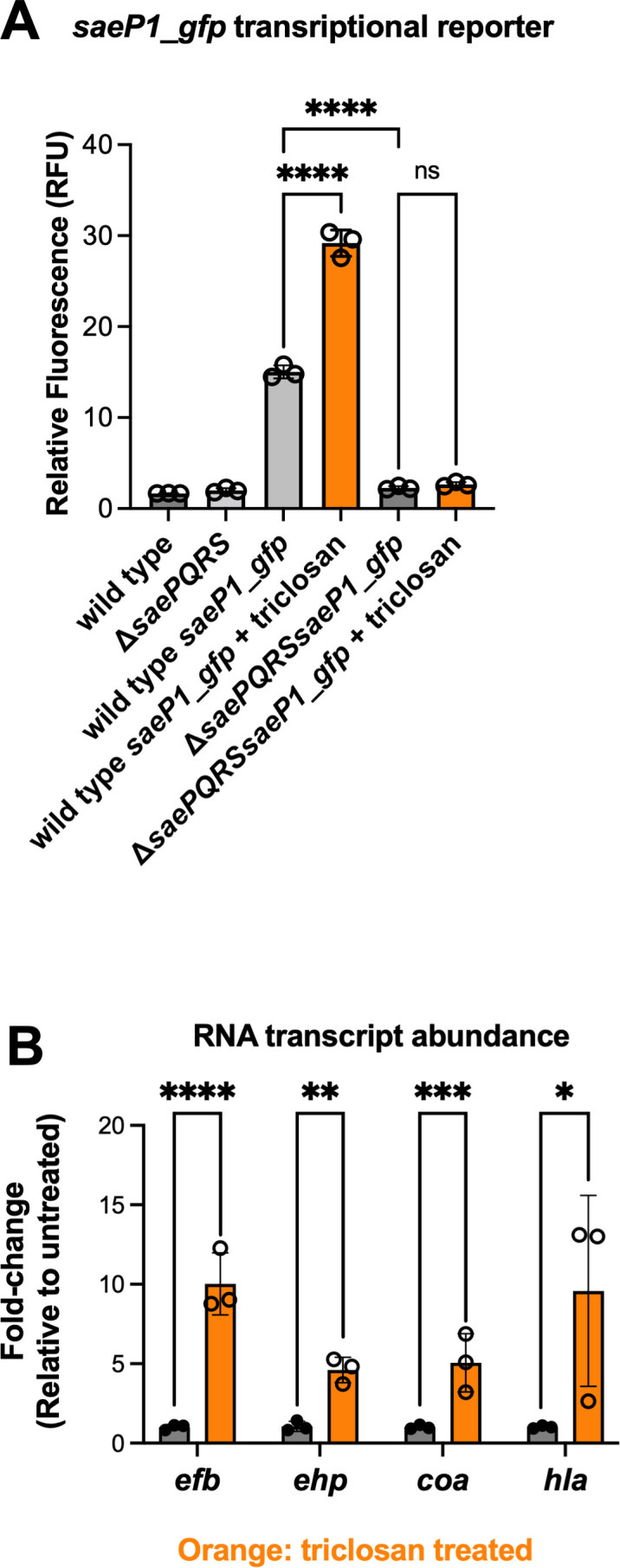
*saeP1* promoter activity is stimulated by triclosan in a *sae*-dependent manner. (**A**) Impact of triclosan treatment on *saeP1* transcriptional activity. Exponential phase cells of the wild-type (JMB 1100) or Δ*saePQRS* (JMB 9891) strains with or without the *saeP1_gfp* transcriptional reporter (pOS_*saeP1_gfp*) were cultured in TSB supplemented with or without 200 ng mL^−1^ triclosan before relative fluorescence was determined. Sample means were compared using one-way ANOVA, and pair-wise comparisons were conducted using *post hoc* Tukey’s multiple comparison tests. Significance levels are as follows: **** represents *P*-values ≤ 0.0001 and ns signifies not significant. (**B**) The impact of triclosan treatment on the abundances of RNAs coded by SaeRS-regulated genes. The wild-type strain was cultured with and without 5 ng mL^−1^ triclosan before RNAs were isolated and quantified. The orange and gray bars indicate samples that were or were not treated with triclosan, respectively. Student’s t-tests were used to compare the fold changes of the treated and non-treated samples, and *, **, ***, and **** represent *P*-values of ≤0.05, ≤0.01, ≤0.001, and ≤0.0001, respectively. The data presented in each panel represent the average of biological triplicates, with the standard deviations shown.

We tested the hypothesis that triclosan, which inhibits FASII-dependent bacterial fatty acid synthesis, would be a modulator of SaeRS regulatory output (30, 31). We cultured the WT strain harboring a *saeP1::gfp* transcriptional reporter and added 200 ng mL^−1^ (0.7 µM) triclosan to the media and incubated for 2 hours before quantifying GFP fluorescence. This concentration of triclosan was chosen because a study by MacIsaac et al. found that the median concentrations of triclosan in the urine of hospital employees who were users of triclosan-containing hand soaps and persons who use toothpaste containing triclosan were 206 and 146 ng mL^−1^, respectively (58). We observed that the triclosan-treated cells displayed a roughly twofold increase in *saeP1* transcriptional activity (Fig. 2A). A Δ*saePQRS* mutant strain did not show significant changes in *saeP1* promoter activity upon triclosan treatment, demonstrating that the increased *saeP1* transcriptional activity required SaeRS.

SaeRS acts as a transcriptional activator for the expression of many genes coding excreted virulence factors, including hemolysins (*hla*), proteins that interact with fibrinogen (*coa*, *efb*), or immune response modulators (*ehp*) (59). We treated WT cells with 5 ng mL^−1^ (18 nM) triclosan and monitored RNA abundances using quantitative PCR (qPCR). The abundances of the SaeRS-regulated RNAs examined significantly increased upon treatment with triclosan (Fig. 2B). We used a lower concentration of triclosan for these experiments to decrease the probability that triclosan treatment would significantly impede cell growth and, thereby, cause growth phase-dependent transcriptional changes. These data demonstrate that co-culture with triclosan increases transcription of SaeRS-regulated genes.

### Triclosan-dependent activation of Sae is independent of SaeP, SaeQ, and cellular respiration

**Fig 3:**
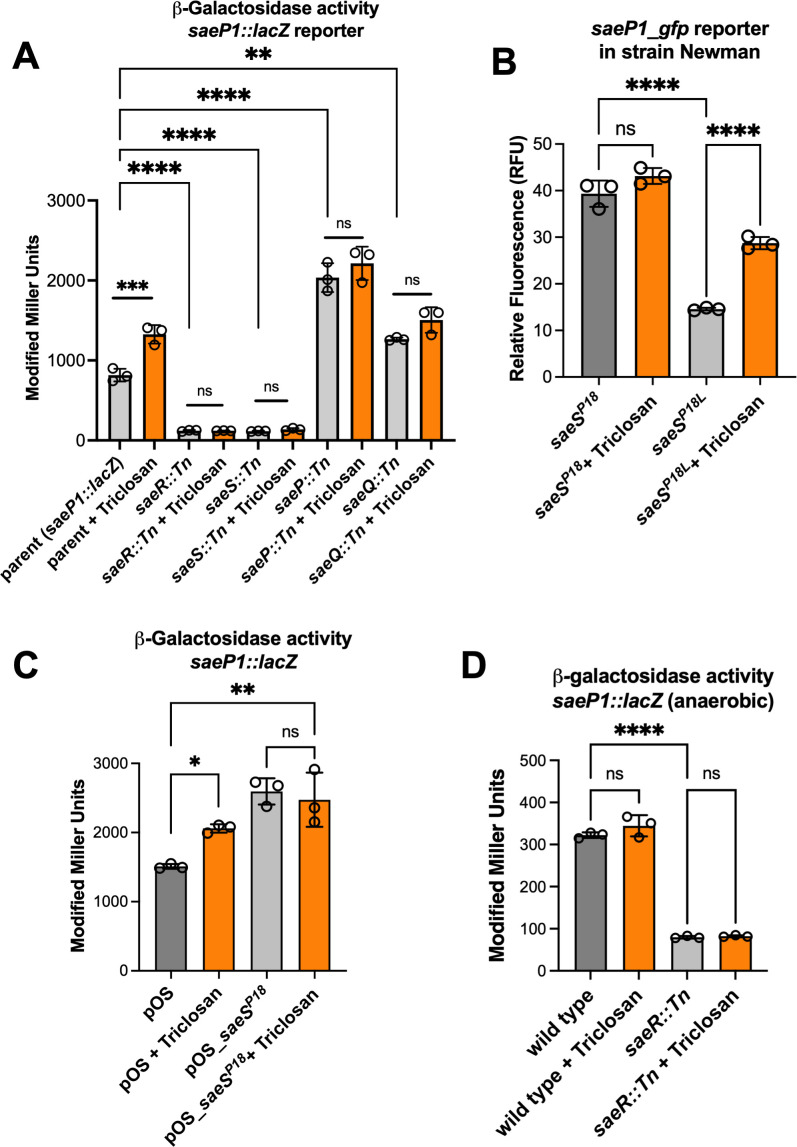
Role of the *sae* locus on the transcriptional response to triclosan. (**A**) Beta-galactosidase activity of the parent (JMB 9709), *saeR::Tn* (JMB 9727), *saeS::Tn* (JMB 10884), *saeP::Tn* (JMB 9728), and *saeQ::Tn* (JMB 10883) strains containing the *saeP1::lacZ* construct. Strains were cultured to logarithmic phase in TSB medium supplemented with (orange bars) or without (gray bars) 200 ng mL^−1^ triclosan. Sample means were compared using two-way ANOVA, and pair-wise comparisons were conducted using *post hoc* Tukey’s multiple comparison tests. Significance levels are as follows: *, **, ***, and **** represent *P*-values of ≤0.05, ≤0.01, ≤0.001, and ≤0.0001, respectively. ns signifies not significant. (**B**) *S. aureus* strain Newman containing the *saeS^P18^* allele is not stimulated by triclosan, whereas strain Newman containing the *saeS^L18^* allele is. Fluorescence was quantified from the Newman strains containing either the *saeS^P18^* (JMB 1422) or *saeS^L18^* alleles (JMB 8263), and the *saeP1_gfp* transcriptional reporter was determined after culture with (orange bars) and without (gray bars) 200 ng mL^−1^ triclosan. (**C**) Constitutive expression of the *saeS^P18^* allele induces *saeP1* promoter activity in a triclosan-independent manner in the LAC wild-type strain. The β-galactosidase activity of the LAC wild-type strain containing the *saeP1::lacZ* transcriptional reporter (JMB 9709) carrying pOS (empty vector) or pOS*_saePQRS^P18^* was quantified following 200 ng mL^−1^ triclosan treatment. (**D**) Triclosan does not stimulate SaeRS during anaerobic growth. Beta-galactosidase activity was monitored in *saeP1::lacZ* (JMB 9709) and the *saeP1::lacZ saeR::Tn* (JMB 9727) strains after anaerobic culture in the presence (orange bars) or absence (gray bars) of 200 ng mL^−1^ triclosan. The data displayed in each panel represent the average of biological triplicates with the standard deviations shown. For panels B, C, and D, sample means were compared using one-way ANOVA, and pair-wise comparisons were conducted using *post hoc* Tukey’s multiple comparison test. Significance levels are as follows: *, **, ***, and **** represent *P*-values of ≤0.05, ≤0.01, ≤0.001, and ≤0.0001, respectively. ns signifies not significant.

The SaeRS regulatory system is comprised of a DNA-binding response regulator (SaeR) and a membrane-associated histidine kinase (SaeS). SaeS also interacts with the auxiliary SaePQ protein complex, which stimulates the phosphatase activity of SaeS (14). We introduced individual insertional inactivation mutations in each of the four open reading frames within the *sae* locus into the *saeP1::lacZ* strain and quantified β-galactosidase activity after culture with and without triclosan. As noted previously, the *saeP1::lacZ* strain exhibited a significant increase in SaeRS activity when treated with triclosan (Fig. 3A). The strains containing the *saeR::Tn* or *saeS::Tn* mutations displayed significantly decreased β-galactosidase expression when compared to the parent and were not stimulated by the addition of triclosan (Fig. 3A). Conversely, the strains containing the *saeP::Tn* and *saeQ::Tn* mutations displayed significantly increased β-galactosidase expression, which was likely due to decreased stimulation of SaeS phosphatase activity. However, this stimulation was not further elevated with triclosan treatment. These data demonstrate that the increased SaeRS output upon triclosan treatment requires a functional and responsive SaeRS system. They are consistent with published data indicating that strains lacking SaePQ have decreased phosphatase activity, resulting in a SaeRS system that cannot be further stimulated (28).

The *S. aureus* LAC strain carries the *saeS^L18^* allele, which is standard for *S. aureus* strains. The *S. aureus* Newman strain carries a *saeS^P18^* allele, which codes for a SaeS variant that has constitutive kinase activity, resulting in an increased pool of phosphorylated SaeR (13, 16, 59). We hypothesized that strain Newman would display increased *saeP1* promoter activity and not respond to triclosan treatment. For this study, we utilized the parent Newman strain (*saeS^P18^*) and a Newman strain that had the *saeS^P18^* allele replaced with *saeS^L18^*. As previously reported, the Newman strain containing the *saeS^P18^* allele had increased *saeP1* transcriptional activity compared to the Newman strains carrying the *saeS^L18^* allele (Fig. 3B) (47). As hypothesized, the transcriptional activity of the *saeP1* promoter was stimulated by triclosan in the strain carrying the *saeS^L18^* allele but not in the strain carrying the *saeS^P18^* allele.

We created an *S. aureus* USA300_LAC wild-type strain that constitutively expressed the *saeS^P18^* allele via a plasmid (pOS_*saePQRS^P18^*) (60). The wild-type LAC strain carrying pOS_*saePQRS^P18^* had increased *saeP1* transcriptional activity compared with the strain carrying the empty vector (pOS), demonstrating that the *saeS^P18^* allele is dominant. Whereas the LAC strain with an empty vector was stimulated by triclosan, the LAC strain carrying the *saeS^P18^* allele in multi-copy was not (Fig. 3C).

We previously discovered that the lack of respiration alters SaeRS output (60). We tested whether respiration was required for triclosan-dependent stimulation of *saeP1* transcriptional activity. Cultures of the *saeP1::lacZ* transcriptional reporter strain were grown anaerobically without a terminal electron acceptor and treated with triclosan in an equivalent fashion to the aerobic experiments reported above. Triclosan-treated cultures did not show significant changes in *saeP1* promoter activity during anaerobic growth, demonstrating that the stimulation of *saeP1* transcriptional activity by triclosan requires respiration under the growth conditions examined (Fig. 3D).

### A strain with a *fabI* allele providing triclosan resistance has decreased SaeRS activation and is not simulated by triclosan

**Fig 4:**
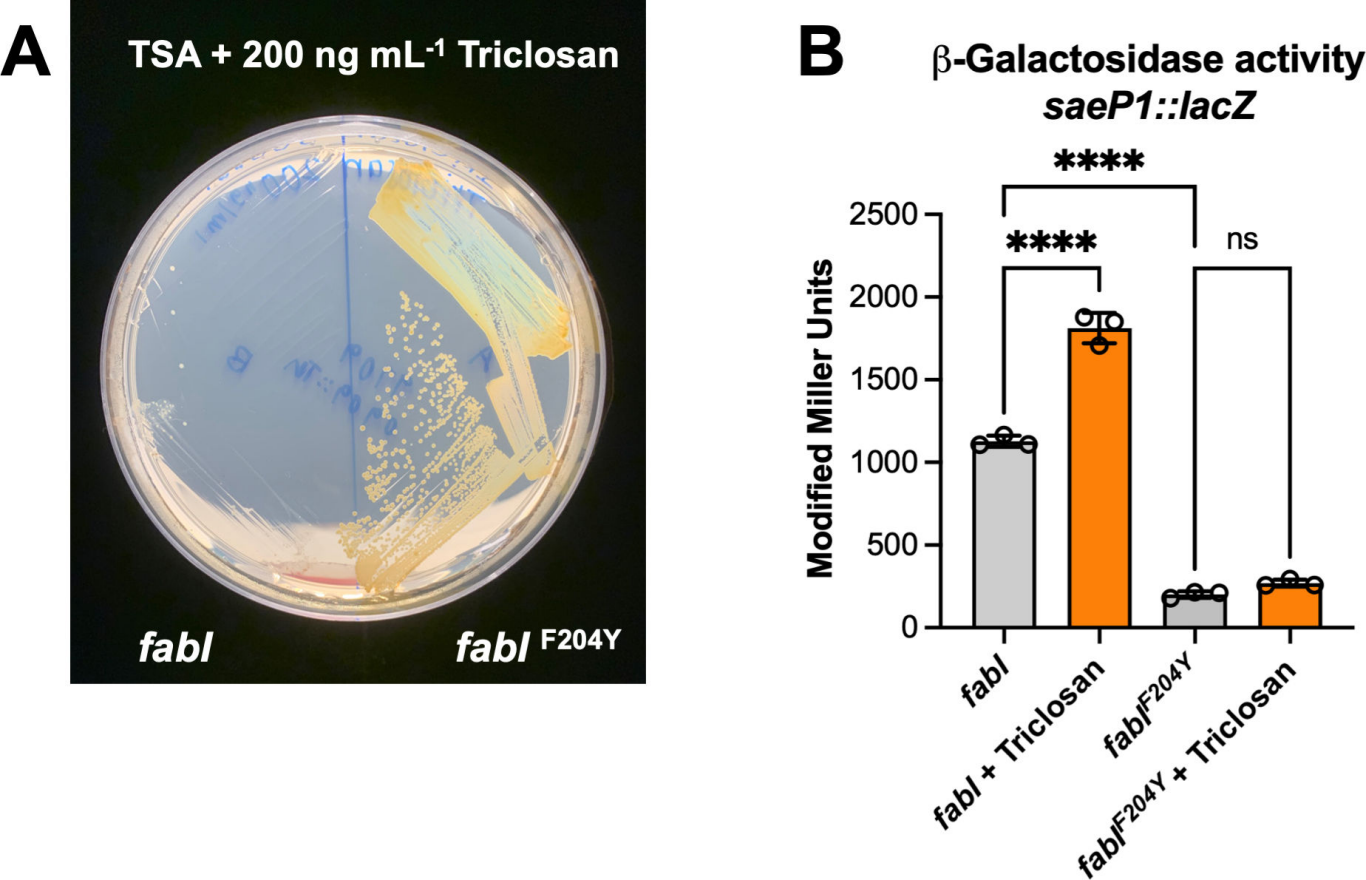
Stimulation of *saeP1* promoter activity by triclosan requires inhibition of fatty acid synthesis. (**A**) Isolation streaks of strains containing either the wild-type copy of *fabI* (JMB11603) or the *fabI*^F204Y^ allele (JMB11601) on TSB medium containing 200 ng mL^−1^ triclosan. (**B**) SaeRS activity is not stimulated by triclosan in a strain carrying the *fabI*^F204Y^ allele. Strains containing *saeP1::lacZ* and the wild-type allele of *fabI* (JMB 11603) or the *fabI*^F204Y^ allele (JMB 11601) were cultured to logarithmic growth phase, treated with 200 ng mL^−1^ triclosan (orange bars), or not (gray bars) before beta-galactosidase activity was quantified. The data displayed in panel B are the average of biological triplicates, with the standard deviations shown. Sample means were compared using one-way ANOVA, and pair-wise comparisons were conducted using *post hoc* Tukey’s multiple comparison test. Significance levels are as follows: **** represents *P*-values of ≤0.0001 and ns signifies not significant.

We tested the hypothesis that triclosan activation of SaeRS requires an allele of *fabI* that codes for an enzyme that triclosan can inhibit. To this end, we plated *S. aureus* LAC on solid media containing triclosan and isolated a strain with a second-site suppressor mutation that increased triclosan tolerance (Fig. 4A). Sequencing of the *fabI* locus revealed that the strain with increased triclosan tolerance harbored a *fabI^F204Y^* allele. The F204 residue of FabI was previously shown to be required for triclosan binding (61, 62), and it has previously been shown that resistance to triclosan can be gained by *S. aureus* through mutations in the *fabI* gene (63). We linked a transposon (SAUSA300_0909::Tn) to the *fabI^F204Y^* allele and used this strain to transduce the *fabI^F204Y^* allele into the *saeP1::lacZ* reporter strain. As expected, we isolated two classes of strains containing the transposon: one with the *fabI^F204Y^* allele providing increased tolerance to triclosan and another with wild-type *fabI* that had a tolerance to triclosan that was indistinguishable from that of *S. aureus* LAC.

Using these isogenic strains, we discovered that *saeP1* transcriptional activity was not stimulated by triclosan in the strain harboring the *fabI^F204Y^* allele, but it was in the strain with the strain containing the wild type *fabI* allele (Fig. 4B). We also determined that the basal level of *saeP1* transcriptional activity was lower in the strains with the *fabI^F204Y^* allele. These data are consistent with the hypothesis that triclosan-dependent stimulation of SaeRS requires a strain with a *fabI* allele that codes an enzyme that can be inhibited by triclosan, resulting in decreased FabI-directed fatty acid synthesis.

### The accumulation of fatty acids nullifies triclosan activation of SaeRS

**Fig 5:**
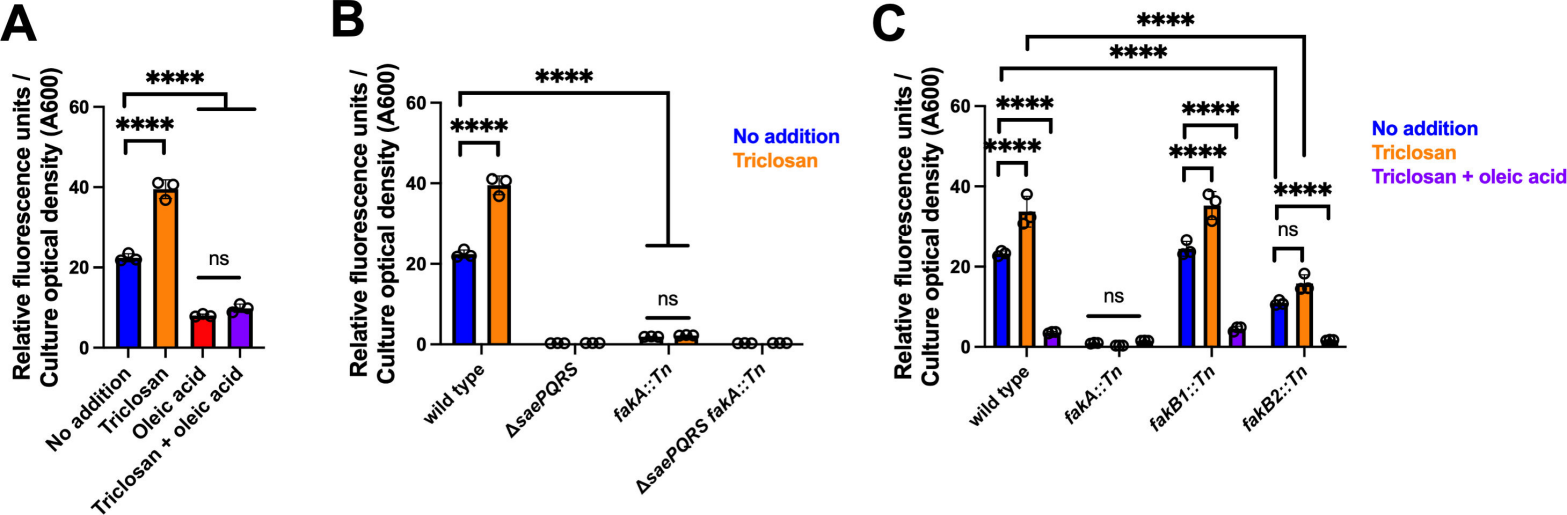
Accumulation of fatty acids nullifies the effect of triclosan treatment on SaeS stimulation. (**A**) Impact of triclosan and oleic acid treatment on *saeP1* transcriptional activity. Exponential phase cells of the wild-type (WT; JMB 1100) containing the *saeP1_gfp* transcriptional reporter (pOS_*saeP1_gfp*) were cultured in TSB until mid-logarithmic phase growth, and the media were supplemented with or without 200 ng mL^−1^ triclosan and/or 0.1% oleic acid before fluorescence was quantified. (**B**) The wild-type, Δ*saePQRS* (JMB 9891), *fakA::Tn* (JMB 13928), Δ*saePQRS fakA::Tn* (JMB 13931) strains containing the *saeP1_gfp* transcriptional reporter (pOS_*saeP1_gfp*) were cultured to mid-logarithmic phase growth, and the media were supplemented with or without 200 ng mL^−1^ triclosan before fluorescence was quantified. (**C**) The wild-type, *fakA::Tn*, *fakB1* (JMB 13929), and *fakB2* (JMB 13930) containing the *saeP1_gfp* transcriptional reporter (pOS_*saeP1_gfp*) were cultured to mid-logarithmic phase growth, and the media were supplemented with or without 200 ng mL^−1^ triclosan or 0.1% oleic acid before fluorescence was quantified. The data displayed are the average of biological triplicates, with the standard deviations shown. Sample means were compared using one-way (panel A), two-way (panel B), or three-way (panel C) ANOVA, and pair-wise comparisons were conducted using *post hoc* Tukey’s multiple comparison tests. Significance levels are as follows: *, **, ***, and **** represent *P*-values of ≤0.05, ≤0.01, ≤0.001, and ≤0.0001, respectively. ns signifies not significant.

We tested the hypothesis that fatty acid inhibition of SaeRS would be dominant and would nullify the effect of triclosan on SaeRS activity. We cultured the WT strain containing the *saeP1* transcriptional reporter in the presence or absence of triclosan and supplemented the growth medium with or without 0.01% oleic acid. Again, adding oleic acid decreased *saeP1* transcriptional activity (Fig. 1A and 5A). The presence of triclosan increased *saeP1* activity, but consistent with our hypothesis, this phenotype was nullified by the presence of oleic acid.

Longer-chain fatty acids can be phosphorylated in the cytosol by the kinase FakA (also called VrfB) in a process facilitated by one of two isoforms of the FakB fatty acid binding proteins (FakB1 or FakB2) (25, 64). The FakB1 isoform displays a preference for saturated fatty acids, and FakB2 prefers unsaturated (64, 65). FakA was previously described as an activator of SaeRS (26), which led to the discovery that supplementing the growth medium with exogenous fatty acids results in decreased SaeRS output (26). We cultured the WT and *fakA::Tn* mutant and isogeneic Δ*saePQRS* mutants containing the *saeP1* transcriptional reporter in the presence and absence of triclosan. As previously reported elsewhere, the transcriptional activity of *saeP1* was greatly reduced in the *fakA::Tn* strain (26). Whereas triclosan increased *saeP1* transcriptional activity in the WT, it did not in the *fakA::Tn* mutant (Fig. 5B). The strains lacking *saePQRS* did not respond to triclosan. Further analysis found that *saeP1* transcriptional activity was unchanged in the *fakB1::Tn* strain and decreased in the *fakB2::Tn* mutant (Fig. 5C). The finding that *saeP1* activity was decreased in the *fakB2* mutant, but not the *fakB1* mutant, suggests that FakB2 is the primary isoform required for incorporation of fatty acids into phospholipids under the growth conditions examined (Fig. 5C). Triclosan addition significantly increased *saeP1* activity in the *fakB1* mutant but not in the *fakB2* mutant, again consistent with FakB2 being the primary fatty acid-binding protein under the conditions examined.

### Triclosan treatment alters the quantity and composition of free fatty acids in *S. aureus* cells

**Fig 6:**
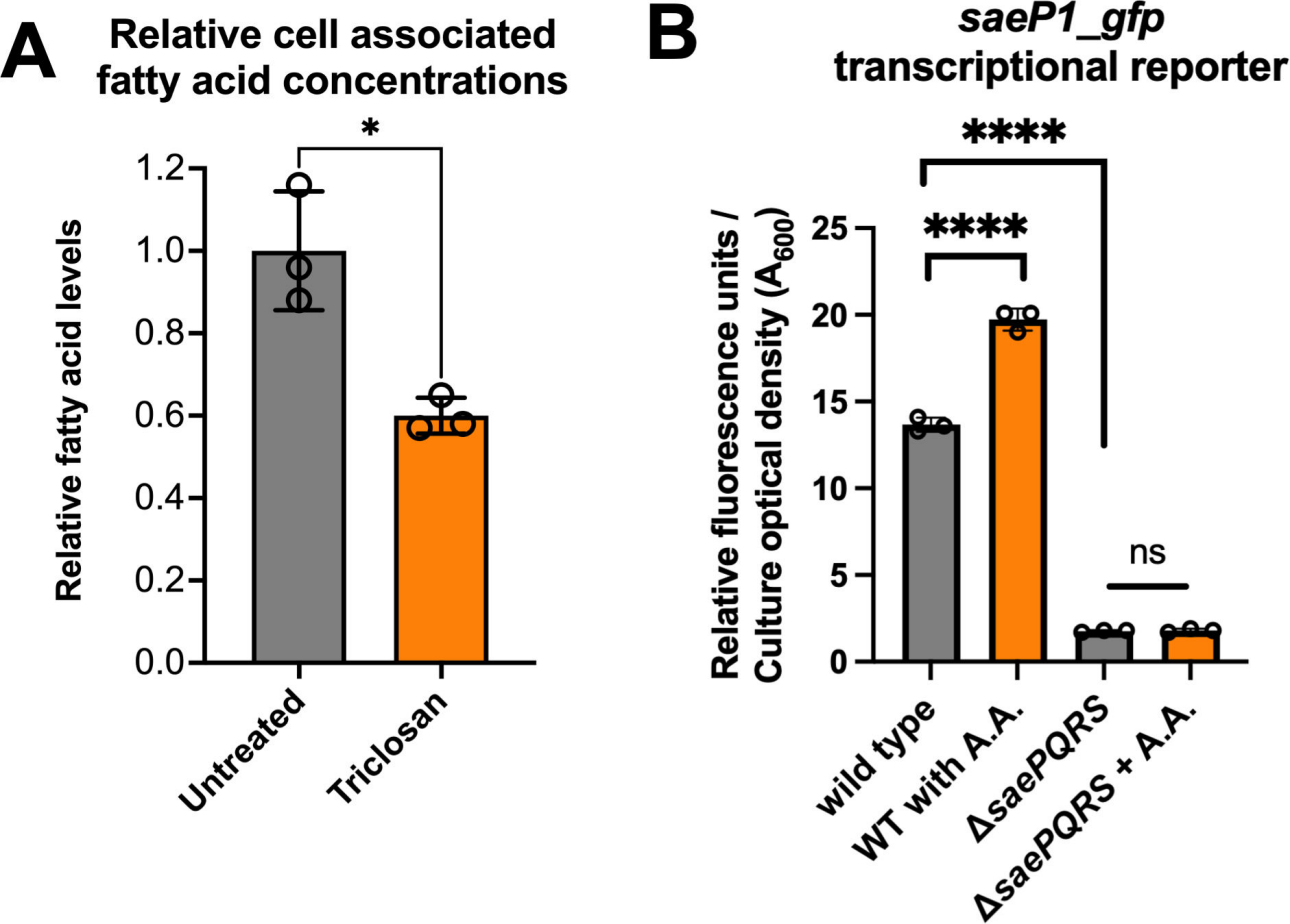
Triclosan treatment decreases the concentration of cell-associated fatty acids, and SaeS is stimulated by arachidic acid. (**A**) The wild-type strain (JMB 1100) was cultured in the presence and absence of 5 ng mL^−1^ triclosan, and fatty acids were isolated and quantified. Student’s t-tests were conducted on the data, and * represents a *P*-value ≤ 0.05. (**B**) Exponential phase cells of the wild-type (WT) or Δ*saePQRS* (JMB 9891) strains harboring a *saeP1_gfp* transcriptional reporter (pOS_*saeP1_gfp*) were cultured in TSB supplemented with or without 200 µM arachidic acid (A.A.) before fluorescence was quantified. Relative fluorescence for biological triplicates is displayed, and standard deviations are shown. Sample means were compared using one-way ANOVA, and pair-wise comparisons were conducted using *post hoc* Tukey’s multiple comparison test. Significance levels are as follows: **** represents a *P*-value of ≤0.0001 and n.s. signifies not significant.

We tested the hypothesis that triclosan treatment alters the titers of fatty acids in *S. aureus* cells. We cultured WT cells with and without triclosan and then isolated and quantified the total free fatty acids. The concentration of total free (nonesterified) cell-associated fatty acids was significantly decreased upon culturing with triclosan (Fig. 6A). The assay used allows for the quantification of nonesterified fatty acids ≥ eight carbons.

We next used unbiased lipidomics to determine the concentrations of individual longer-chain fatty acids. The concentrations of lauric acid (12:0), myristic acid (14:0), and arachidic acid (20:0) were significantly increased in triclosan-treated cells (Table 3). The concentrations of palmitic acid (16:0) and lignoceric acid (24:0) were significantly decreased in triclosan-treated cells. These data suggest that the overall abundance of free fatty acids with at least eight carbons decreases with triclosan treatment, but the abundances of a few individual fatty acids also increase upon triclosan treatment.

**TABLE 3:** Abundances of fatty acids in *S. aureus* after treatment with triclosan

Fatty acid	Untreated^*a*^	Triclosan treated^*b*^	Fold-increase upon treatment	*P-*value^*c*^
C6:0	0.5 ± 0.2	0.6 ± 0.2	1.2	0.55
C8:0	0.3 ± 0.3	0.4 ± 0.2	1.3	0.66
C10:0	0.7 ± 0.3	1.2 ± 0.3	1.8	0.08
C12:0	282 ± 14	331 ± 28	1.1	0.05
**C14:0**	**34.0 ± 1.4**	**178 ± 45**	**5.2**	**0.01**
C14:1Δ5	7.5 ± 1.5	7.3 ± 0.4	1.0	0.86
**C16:0**	**1234 ± 27**	**1158 ± 19**	**0.9**	**0.01**
C16:1Δ7	9.9 ± 1.9	12 ± 1	1.2	0.18
C18:0	928 ± 20	913 ± 11	1.0	0.30
C18:1Δ9	15.7 ± 4.8	17.5 ± 2.7	1.1	0.59
C18:1Δ7	5.7 ± 0.6	5.8 ± 1.3	1.0	0.89
C18:2Δ6	2.9 ± 0.1	4.3 ± 1.3	1.5	0.14
C18:3Δ6	3.5 ± 1.3	3.9 ± 1.0	1.1	0.71
C18:3Δ3	0.6 ± 0.1	1.0 ± 0.6	1.6	0.31
**C20:0**	**123 ± 5**	**418 ± 80**	**3.4**	**0.00**
C20:1Δ9	5.1 ± 0.6	5.9 ± 1.3	1.2	0.38
C20:2Δ6	4.0 ± 1.4	2.5 ± 0.8	0.6	0.17
C20:3Δ6	3.4 ± 0.9	4.3 ± 0.6	1.3	0.23
C20:4Δ6	2.9 ± 0.9	3.3 ± 1.5	1.1	0.66
C20:3Δ3	0.3 ± 0.1	0.3 ± 0.2	1.3	0.55
C20:5Δ3	0.4 ± 0.2	0.4 ± 0.1	0.9	0.75
C22:0	114 ± 5	136 ± 20	1.2	0.13
C22:1Δ9	0.5 ± 0.1	0.8 ± 0.1	1.6	0.06
C22:2Δ6	0.3 ± 0.2	0.3 ± 0.1	1.0	0.93
**C24:0**	**56.9 ± 1.7**	**52.5 ± 0.9**	**0.9**	**0.02**
C22:6Δ3	4.5 ± 3.6	3.9 ± 0.3	1.1	0.54

^*a*^ µg compound per mg of cells. Data present the average and standard deviation of biological triplicates.

^*b*^ Cultures were treated with 5 ng mL^−1^ triclosan.

^*c*^ *P-*value determined by conducting a student’s two-tailed t-tests. Significant differences are shown in bold.

It was previously demonstrated that stearic acid (18:0), unlike other shorter-chain fatty acids tested, increased SaeRS output (28). We tested the hypothesis that arachidic acid (A.A.) (20:0), which accumulated upon triclosan treatment, alters *saeP1* transcription. We cultured the WT and Δ*saePQRS* strains containing the *saeP1_gfp* transcriptional reporter with and without A.A. and quantified *gfp* expression. Growth with A.A. increased *saeP1* promoter activity in the WT but not in the Δ*saePQRS* strain (Fig. 6B).

### Growth with triclosan or arachidic acid increases the ability of *S. aureus* to cause SaeRS-dependent cytotoxicity

**Fig 7:**
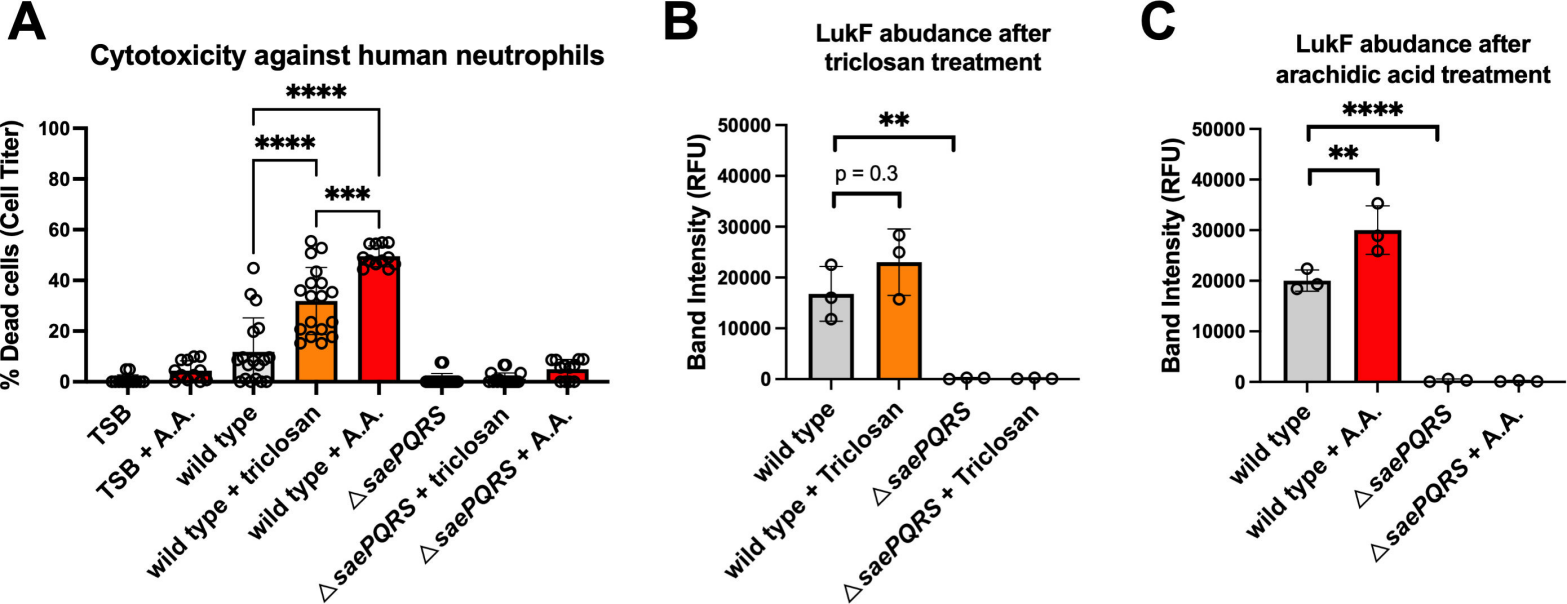
Treatment of *S. aureus* with either triclosan or arachidic acid increases neutrophil cytotoxicity and accumulation of LukF. (**A**) Triclosan or arachidic acid treatment increases the ability of *S. aureus* to cause human neutrophil cytotoxicity. The wild-type strain (JMB 1100) was cultured in TSB to exponential phase and treated with 200 mg mL^−1^ triclosan (orange bars), 200 µM arachidic acid (A.A.) (red bars), or no addition (gray bars). Cell supernatants were isolated and combined (0.4%) with human neutrophils (4–6 donors), and cell viability was evaluated using CellTiter (Promega). Sample means were compared using two-way ANOVA, and pair-wise comparisons were conducted using *post hoc* Tukey’s multiple comparison test. Significance levels are as follows: *, **, ***, and **** represent *P*-values of ≤0.05, ≤0.01, ≤0.001, and ≤0.0001, respectively. Triclosan (**B**) or arachidic acid (**C**) treatment increases LukF expression. The wild-type strain was cultured in TSB to exponential phase with 200 mg mL^−1^ triclosan (orange bars), 200 µM arachidic acid (A.A.) (red bars), or no addition. Cell supernatants were isolated, proteins were precipitated, and total LukF was quantified using Western blotting. Band intensity is the average of biological triplicates (Fig. S1), and standard deviations are shown. Sample means were compared using one-way ANOVA, and pair-wise comparisons were conducted using *post hoc* Tukey’s multiple comparison test. Significance levels are as follows: *, **, ***, and **** represent *P*-values of ≤0.05, ≤0.01, ≤0.001, and ≤0.0001, respectively.

SaePQRS controls the expression of many secreted virulence factors, including leukocidins, which cause cytotoxicity and lyse neutrophils (66). We cultured the WT and Δ*saePQRS* strains in TSB media with or without triclosan or A.A. and isolated the cell-free culture supernatants. We then combined TSB, TSB with A.A., or spent culture media with human neutrophils and monitored cell death. The spent media supernatants isolated from the WT strain cultured with either triclosan or A.A. killed a significantly higher proportion of the neutrophils than the spent culture media from the Δ*saePQRS* strain treated with triclosan or A.A. (Fig. 7A). These data are consistent with the hypothesis that treatment of WT cells with either triclosan or A.A. increases leukocidin production in a SaeRS-dependent manner.

We further examined whether the accumulation of the leukocidin LukF was altered in the cell-free lysates isolated from the WT or Δ*saePQRS* strains. We separated the excreted proteins using SDS-PAGE and then conducted Western blotting to quantify LukF. LukF was robustly detected in the WT strain, but as expected, titers were significantly decreased in the Δ*saePQRS* strain (Fig. 7B and C; Fig. S1). The abundance of LukF increased upon treatment with triclosan or A.A.; however, the difference in production did not reach statistical significance upon triclosan treatment.

## DISCUSSION

Previous work by others demonstrated that strains defective in incorporating fatty acids into phospholipids have decreased output from the SaeRS regulatory system (26). Further analyses found that supplementing the growth medium with fatty acids of various chain lengths also inhibited SaeRS-dependent signaling (28). The transmembrane portion of the SaeS sensor kinase was required for inhibition by fatty acids, suggesting that fatty acid titers directly alter SaeS activity, but the mechanism of inhibition is currently unknown (28). These findings are significant because the SaeRS system directly controls the transcription of numerous genes that code for toxins and virulence factors and indirectly controls the expression of others (14). Triclosan is a widely used antimicrobial and an inhibitor of fatty acid synthesis in bacteria. It binds to the enoyl-acyl carrier protein reductase FabI and locks it in an inactive tetrameric complex, thereby inhibiting FASII-dependent fatty acid synthesis (31, 67). Herein, we tested the hypothesis that inhibiting fatty acid synthesis in *S. aureus* would decrease cellular free fatty acid titers and thereby increase SaeRS output.

**Fig 8:**
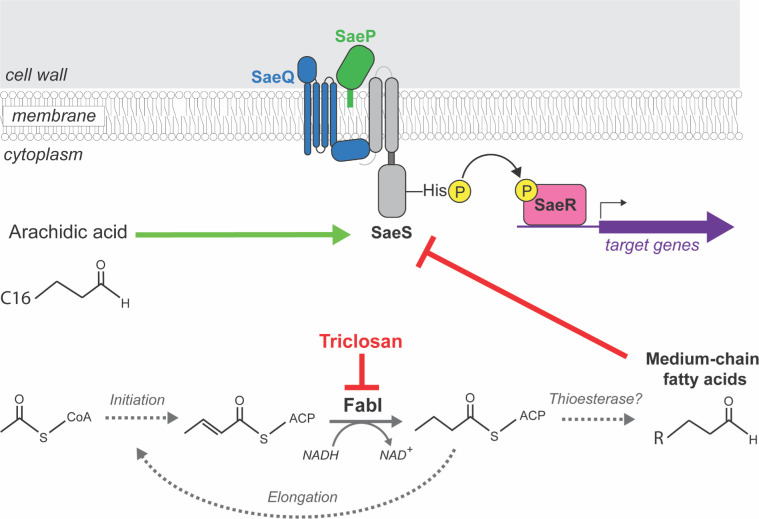
A working model for the influence of triclosan upon SaeRS-dependent virulence factor expression in *S. aureus*. Long-chain free fatty acids stimulate the SaeS histidine kinase, resulting in a decreased concentration of phosphorylated SaeR and, subsequently, lower expression of target genes. Triclosan inhibits the enoyl-acyl carrier protein FabI and decreases the accumulation of soluble fatty acids. Triclosan treatment also increases the accumulation of arachidic acid, which positively stimulates SaeS. Stimulation of SaeS increases the titers of phosphorylated SaeR, resulting in increased transcription of genes that code for virulence factors.

The data presented in this manuscript led to a working model displayed in Fig. 8. Upon growth with triclosan, FabI is inhibited, decreasing the pool of fatty acids bound to acyl-carrier protein. Fatty acids are liberated from the acyl-carrier protein by a currently unknown mechanism, possibly due to an unidentified thioesterase. Triclosan treatment decreases total medium- and long-chain fatty acid accumulation and a concomitant increase in arachidic acid (C20:0) accumulation. The alterations in fatty acid titers stimulate SaeS kinase activity, increasing phosphorylated SaeR. Increased phosphorylated SaeR promotes transcription of positively regulated genes of the SaeRS regulon. Activation of the SaeRS regulon increases the production of virulence factors that contribute to pathogenesis.

Several results presented are consistent with the hypothesis that altering fatty acid titers modulates SaeRS output. First, treatment with triclosan increased transcription of genes directly regulated by SaeRS (Fig. 2). Strains lacking functional SaeP or SaeQ, which stimulate SaeS phosphatase activity, had increased transcription of class I target genes, which require a high level of phosphorylated SaeR (14). We demonstrated that SaeRS transcriptional output is increased in the *saeP::Tn* and *saeQ::Tn* mutants and that SaeRS output was not further stimulated by triclosan (Fig. 3A). Similarly, *S. aureus* strains containing the *saeS^L18P^* allele, which codes for a variant with decreased phosphatase activity resulting in a high titer of phosphorylated SaeR (13, 16), had increased transcriptional activity, and transcription was not further stimulated by triclosan (Fig. 3B and C). Supplementing the growth medium with oleic acid decreased SaeRS activity and nullified the effect of triclosan (Fig. 5A). Likewise, a *fakA* mutant, which is defective in generating fatty acids into phospholipids resulting in fatty acid accumulation (64), had decreased SaeRS output and nullified the effects of triclosan (Fig. 5B). Taken together, these data suggest that triclosan treatment has a stimulatory effect on SaeS, resulting in increased titers of phosphorylated SaeR; however, additional biochemical analyses are necessary to verify whether the phosphorylation status of SaeR is altered upon triclosan treatment and to quantify these differences.

We isolated a strain with a *fabI^F204Y^* allele that had increased tolerance to growth with triclosan. Importantly, SaeRS output in the *fabI^F204Y^* mutant was not altered upon exposure to triclosan, suggesting that triclosan was stimulating SaeRS via inhibition of FabI (Fig. 4B). The *fabI^F204Y^* mutant had lower SaeRS output than a strain with a WT *fabI* allele, suggesting an increased basal rate of fatty acid production. Several strains have been isolated and characterized with mutations at the F204 position, with the *fabI^F204C^* and *fabI^F204S^* alleles being the most common (62, 68–70). To our knowledge, a *fabI^F204Y^* mutant has not been previously described. However, biochemical characterization of the FabI^F204S^ and FabI^F204C^ variants demonstrated increased catalytic efficiencies (*k*_cat_), signifying increased turnover (68, 70). Further biochemical analysis will be necessary to determine whether the FabI^F204Y^ variant also has increased catalytic efficiency.

Treatment of *S. aureus* with triclosan decreased titers of cell-associated fatty acids, but when we quantified individual fatty acids, the concentrations of a couple of saturated fatty acids (C14:0 and C20:0) increased (Fig. 6A; Table 3). The finding that total fatty acids are less abundant upon treatment with triclosan is consistent with fatty acids being synthesized by the FASII complex and inhibition of that complex by triclosan. Growth with triclosan also altered free fatty acid concentrations in *Pseudomonas aeruginosa* (71). Like the data presented in this report, the concentrations of some fatty acids remained unchanged (C16:0) after co-culture, while others increased (C18:0) or decreased (C17:0 and C19:0) in abundance. Unlike reported herein, the authors did not note a significant alteration in total readily extractable lipids when cells were dosed with triclosan. When the phospholipid fatty acyl esters were converted to volatile fatty acids methyl esters (FAME) and quantified, there was a significant decrease in FAME with triclosan but overall maintenance of total fatty acid levels. The fatty acids that had altered abundances in triclosan-treated *S. aureus* were also saturated. It is currently unknown how dosing cells with triclosan alters the pools of free fatty acids in either *P. aeruginosa* or *S. aureus*. Further studies are required to uncover mechanistic details.

The finding that triclosan treatment stimulates SaeRS is significant because triclosan is recalcitrant to biotic and abiotic breakdown, leading to its accumulation in environments (72, 73). Triclosan is added to numerous consumer and medical products, and as a result, it has been demonstrated to accumulate in the human body (74). One study reported that triclosan could be detected in 75% of human study participants, and concentrations of 2.4–3,790 ng mL^−1^ (*n* = 2,517) were detected in urine with a mean triclosan concentration of 13 ng mL^−1^ (75). MacIsaac et al. reported that the median concentration of triclosan in the urine of hospital employees using triclosan-containing products (*n* = 29) was 256 ng mL^−1^ (58). These subjects were physicians and nurses who washed their hands frequently with triclosan-containing hand soaps. For physicians and nurses not using triclosan hand soaps (non-triclosan users) (*n* = 47), the researchers found that the median concentration of triclosan in urine was 46 ng mL^−1^. The same study found that persons who used toothpaste containing triclosan had an average level of 146 ng mL^−1^ triclosan in their urine. Triclosan is commonly found in domestic wastewater and is inefficiently removed by sewage treatment plants, resulting in triclosan dosing to aquatic ecosystems and accumulation in sediment (73, 76, 77). Concentrations of triclosan in surface waters range from 1.4 to 40,000 ng L^−1^ (78).

Although we have demonstrated that triclosan treatment increases SaeRS-dependent gene transcription, which others have shown is vital for expressing many excreted virulence factors, it is unknown if triclosan in the environment or mammalian body would alter *S. aureus* pathogenesis. However, our findings have opened the door for additional studies examining whether treating *S. aureus* with growth-permissive concentrations of triclosan alters tissue colonization or virulence. The frequency of *S. aureus* Newman isolates displaying resistance to 250  ng mL^−1^ triclosan is increased about 100-fold when the growth medium is supplemented with fatty acids (79, 80). The suppressed *S. aureus* strains contained mutations in *accABCD* (acetyl-CoA carboxylase) or *fabD*, which code for enzymes involved in *de novo* FASII fatty acid synthesis. These studies suggest that (i) triclosan-mediated killing of *S. aureus* requires inhibition of fatty acid synthesis and (ii) access to host-derived fatty acids may promote selection for strains that are resistant to FASII inhibitors. Importantly, several of the suppressed *S. aureus* strains were still infective. These studies and others imply that host-derived fatty acids could decrease the need for *de novo* fatty acid synthesis (64, 79, 80). Therefore, the presence of host-derived fatty acids could alter the concentrations of select cytosolic fatty acids, such as arachidic acid, which accumulates upon triclosan treatment and increases SaeRS-dependent gene transcription. Future studies will determine whether alternate FabI inhibitors also stimulate SaeRS (68).

## SUPPLEMENTAL MATERIAL

Fig. S1 aac.01728-24-s0001.pdf Western blots.

ASM does not own the copyrights to Supplemental Material that may be linked to, or accessed through, an article. The authors have granted ASM a non-exclusive, world-wide license to publish the Supplemental Material files. Please contact the corresponding author directly for reuse.
