# Supplementary material for: Treatment of Staphylococcus aureus with environmentally relevant concentrations of triclosan activates SaeRS-dependent virulence factor expression
Source: Antimicrob Agents Chemother. 2025 Jun 18;69(8):e01728-24. doi: 10.1128/aac.01728-24 (PMC12327003; doi:10.1128/aac.01728-24)
Supplement: Fig. S1 — Western blots. [file aac.01728-24-s0001.pdf]

Supplemental information for manuscript:

Treatment of *Staphylococcus aureus* with environmentally relevant concentrations of triclosan activates SaeRS-dependent virulence factor expression.

Jeffrey M. Boyd[1*], Erin E. Price[1], Franklin Roman Rodriguez[1], Natalie Burchat[2], Javiera Norambuena[1], Ashley L. DuMont[3,4], Victor J. Torres[3,4], Harini Sampath[2]

Department of Biochemistry and Microbiology, Rutgers, The State University of New Jersey, New Brunswick, NJ 08901, USA

[1] Department of Biochemistry and Microbiology, Rutgers, The State University of New Jersey, New Brunswick, NJ 08901, USA
[2] Department of Nutritional Sciences, Rutgers, The State University of New Jersey, New Brunswick, NJ 08901, USA
[3] Department of Microbiology, New York University Grossman School of Medicine, Alexandria Center for Life Science, New York, NY 10016.
[4] Department of Host-Microbe Interactions, St. Jude Children's Research Hospital, Memphis, TN 38105

* To whom correspondence should be addressed: jeffboyd@sebs.rutgers.edu,
76 Lipman Drive, New Brunswick, NJ 08901

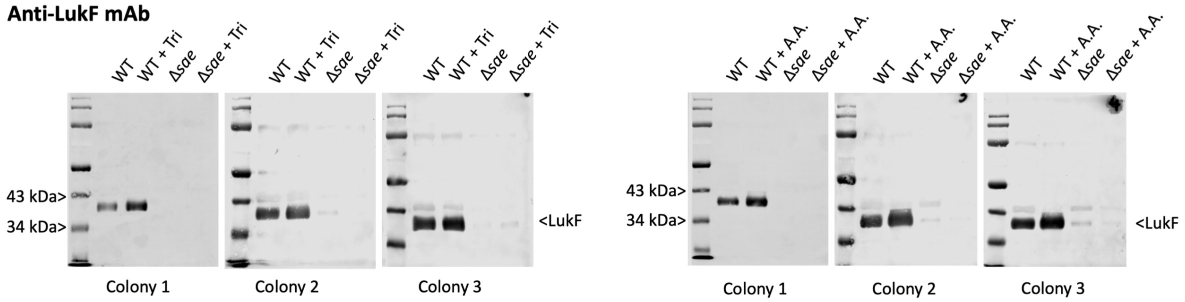

Supplemental Figure 1. Western blots that were used to generate the data displayed in Figure 7. Three independent cultures, inoculated from independent colonies, of the wild type (JMB1100) and Δ*saePQRS* (JMB 9891) mutant strains were cultured in TSB to exponential phase and treated with 200 mg mL^{-1} triclosan (+ Tri), 200 μM arachidic acid (A.A.) (+ A.A.), or no addition. Cell supernatants were isolated, proteins were precipitated, and total LukF was quantified using an anti-LukF monoclonal antibody. Colonies 1, 2, and 3 represent biological triplicates.
